# Rationale for the development of an Alzheimer’s disease vaccine

**DOI:** 10.1080/21645515.2019.1665453

**Published:** 2019-10-22

**Authors:** Ping Kwan, Haruki Konno, Ka Yan Chan, Larry Baum

**Affiliations:** a School of Pharmacy, The Chinese University of Hong Kong, Shatin, Hong Kong SAR, P.R. China; b Sydney School of Veterinary Science, The University of Sydney, Sydney, Australia; c State Key Laboratory of Brain and Cognitive Sciences, The University of Hong Kong, Pokfulam, Hong Kong SAR, P.R. China; d Center for Genomic Sciences, Li Ka Shing Faculty of Medicine, The University of Hong Kong, Pokfulam, Hong Kong SAR, P.R. China; e Department of Psychiatry, Li Ka Shing Faculty of Medicine, The University of Hong Kong, Pokfulam, Hong Kong SAR, P.R. China

**Keywords:** Alzheimer’s disease, amyloid, vaccine, infection, neurodegeneration, dementia

## Abstract

Vaccination traditionally has targeted infectious agents and thus has not heretofore been used to prevent neurodegenerative illness. However, amyloid β (Aβ) or tau, which can act like infectious proteins, or prions, might induce Alzheimer’s disease (AD). Furthermore, evidence suggests that traditional infectious agents, including certain viruses and bacteria, may trigger AD. It is therefore worth exploring whether removing such targets could prevent AD. Although failing to treat AD patients who already display cognitive impairment, Aβ monoclonal antibodies are being tested in pre-symptomatic, at-risk individuals to prevent dementia. These antibodies might become the first AD therapeutics. However, their high cost will keep them out of the arms of the vast majority of patients, who increasingly live in developing countries. Because vaccines produce antibodies internally at much lower cost, vaccination might be the most promising approach to reducing the global burden of dementia.

## 1. Introduction

Alzheimer’s disease (AD) is a progressive neurodegenerative disorder, first reported by Alois Alzheimer around 1907.^1^ AD is the most common cause of dementia and is clinically characterized by memory difficulties, language disturbances, psychological and psychiatric changes, and impairments in activities of daily living.^2^ Pathophysiologically, AD is characterized by the presence of extracellular senile plaques composed mainly of β-amyloid (Aβ) peptides, the presence of intracellular neurofibrillary tangles formed by cytoskeletal protein tau in the neuronal cell body, neuropil threads in dendrites, chronic brain inflammation, oxidative damage, loss of synapses, and selective neuronal cell loss (for example, pyramidal cells in lamina II of the entorhinal cortex and in the CA1 region of the hippocampus).^3^^–5^ About 50 million people have AD, with the number expected to triple by 2050.^6^ The cost of AD is about one trillion US$ per year and is expected to double by 2030.^6^ Although currently there are symptomatic treatments for AD using cholinesterase inhibitors for moderate disease and a glutamatergic partial antagonist for moderately severe disease, these treatments do not stop the progression of dementia.^2^ Thus, it is imperative to investigate or explore further therapeutic options. After reviewing current knowledge of AD and the therapeutic options now being tested, we will present the case for pursuing a vaccination strategy for AD.

**Figure 1.**
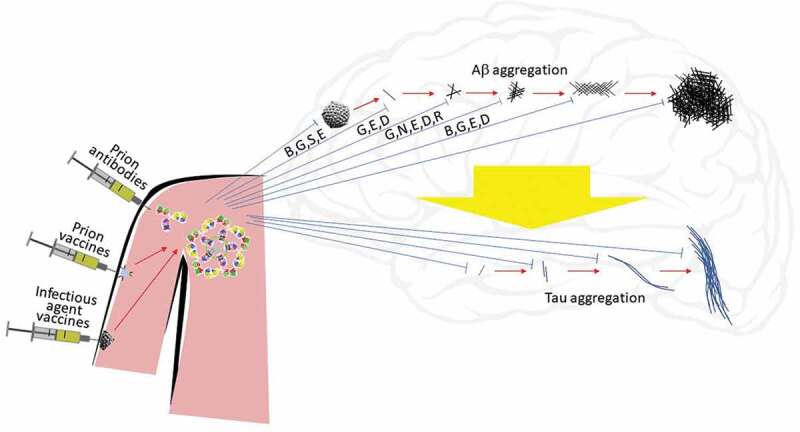
In Alzheimer’s disease (AD), amyloid β protein (Aβ) monomers aggregate into oligomers, protofibrils, fibrils, and amyloid plaques (red arrows, left to right top), possibly inducing tau aggregation (wide yellow arrow) into oligomers, paired helical filaments, and neurofibrillary tangles (left to right bottom). These or associated processes might damage neuronal function and cause dementia. Because Aβ and tau aggregates can induce the spread of Aβ and tau aggregation, they can act like prions. Infection (virus, center top) may stimulate production of Aβ as an innate immune system response. Antibodies (IgG or IgM, center left) may bind and neutralize (blue lines) infectious agents or different stages of aggregation of Aβ or tau, thus possibly preventing or slowing the progression of AD. Antibodies to Aβ or tau (prion antibodies) or to traditional infectious agents such as viruses may be produced artificially and injected, or they may be induced by vaccination (prion vaccines or infectious agent vaccines). Antigens may be fragments of monomers of Aβ or tau or stabilized aggregates (oligomer, with blue disk symbolizing stabilization) or may be fragments of infectious agents. Letter codes indicate specific antibodies known to preferentially bind particular aggregation states of Aβ: B = bapineuzumab, G = GSK933776, S = solanezumab, N = BAN2401, E = gantenerumab, D = aducanumab, R = SAR228810.

## 2. Genetics of Alzheimer’s disease

In a small proportion of cases, Mendelian inheritance leads to an early onset (<60 years) of AD (EO-FAD). However, the vast majority of AD is late-onset (LOAD) and is caused by a variety of environmental and genetic factors.

The major genes that play a role in EO-FAD are amyloid β protein precursor (APP) and the presenilins (PSEN1 and PSEN2).^7^^–13^ Amyloid β (Aβ) peptide, which presenilin helps to cleave from APP, is the major component of the amyloid plaques and cerebral blood vessel deposits characteristic of AD.^14^ Aβ that is 42 amino acids long (Aβ42) aggregates more readily than does Aβ that is 40 amino acids long (Aβ40).^15^ EO-FAD mutations, such as point mutations in APP, PSEN1, or PSEN2, or APP gene duplication, may affect Aβ by different mechanisms: increasing the Aβ42:Aβ40 ratio, increasing the production of Aβ of all lengths, or changing amino acids in Aβ.^16^^–18^ These all increase Aβ aggregation into oligomers and ultimately into amyloid fibrils (Figure 1).^19^ Under some conditions, Aβ aggregates can be cytotoxic.^20,21^ The amyloid cascade hypothesis connects these observations by positing that Aβ is produced and damages neurons, causing dementia.^20,21^ Aβ can act like a prion, aggregating into a form that induces more Aβ to aggregate.^22,23^ Aggregated Aβ can seed Aβ aggregation in other locations and can trigger tau aggregation, which in turn can induce further tau aggregation and spreading to other locations.^22^^–25^

The largest genetic risk factor for LOAD is the APOE ϵ4 allele^26^ as it strongly influences Aβ clearance^27^ or its oligomerization.^28^ One ϵ4 allele increases the risk of AD by approximately 4 times and 2 alleles raise the risk about 12 times.^15^

Polymorphisms in over 20 other genes alter risk for LOAD to a modest extent, with odds ratios ranging from about 0.77 to about 1.23.^15^ These genes function in areas including cholesterol metabolism, immunity, and endocytosis.^15^ Rare variants in some of these and other genes also affect LOAD risk.^15^

## 3. Immunotherapy for AD

### 3.1. Vaccination for AD

If Aβ toxicity causes AD, then removing Aβ from the brain might treat AD. Antibodies might be created to bind Aβ, after which the immune system may then clear the antibody–antigen complex.^29^ Vaccination with Aβ might prime the immune system to target and remove Aβ.^29^ This active immunization strategy has been used against various bacterial, viral, and toxin antigens for over two centuries since Edward Jenner developed vaccination against smallpox.^29^^–31^ Vaccines have achieved enormous health benefits: preventing about six million deaths each year, totally eliminating smallpox, nearly eradicating polio, and greatly reducing measles.^30,31^

Vaccines often contain an antigen, against which the immune system will target a response, and an adjuvant, to stimulate the immune system.^32^ The nature of the antigen may directly affect the effectiveness and even the strategy used in the immunotherapy.^29^ Potential difficulties in targeting Aβ include crossing the blood–brain barrier and inducing an immune response against a protein naturally produced by the body.^29^

Despite such obstacles, Dale Schenk led a project to test Aβ vaccination. In APP transgenic mice that produce high levels of Aβ, vaccination succeeded in preventing the onset of plaque formation in young mice and slowing the progression of plaque formation in older mice.^29^ This led to clinical trial AN-1792, in which 300 AD patients were vaccinated with full length Aβ42.^29^ However, the trial was stopped after some patients suffered meningoencephalitis.^33^ Fifty-nine patients developed the predetermined degree of antibody response, and 18 developed meningoencephalitis (of whom 13 were antibody responders).^33^ During the study follow-up period, five patients died, of whom one (myocardial infarction) was an antibody responder.^33^ Subsequent analysis showed that, in patients who died in the years since the trial and whose brains were studied, plaques and phosphorylated tau decreased, including two patients with nearly complete removal of plaques 14 years after vaccination, compared to non-vaccinated AD patients, though individual cognitive and disability test scores did not significantly improve.^29,33,34^ The continued cognitive deterioration of patients despite extensive plaque clearance suggests that a process, such as spread of tau pathology, continued to harm brain function.^34^ Therefore, very early intervention, perhaps before the initiation of self-propagating spread of tau aggregates, may be needed.^34^ Although AN1792 did not prevent cognitive decline in AD patients, it did demonstrate very long-term effect on Aβ deposition, suggesting the feasibility of vaccination to remove proteins involved in neurodegenerative diseases.^34^

A T-cell response to T-cell epitopes in the middle region of Aβ had occurred.^35^ To prevent this, later trials of Aβ vaccines tended to select antigens that avoided that region by using Aβ fragments,^36^ such as the B-cell epitopes at the amino terminus.^29^

Aβ vaccines subsequently tested in clinical trials have included ABvac40,^37^ CAD106, ACC-001 (vanutide cridiﬁcar), AD01, AD02, AD03, UB-311, V-950, Lu AF20513, and ACI-24.^38^ Some of the trials are ongoing, while others have finished. CAD106, ACC-001, AD01, AD02, AD03, UB-311, and V-950 have reported some results.^38^ CAD106 is being tested in a large trial of cognitively healthy elderly APOE ϵ4 homozygotes.^39^

Only ABvac40 targets the C-terminal end of the Aβ40 peptide.^37^ CAD106, ACC-001, AD01, AD02, UB-311, Lu AF20513, and ACI-24 target the N-terminal of Aβ, with CAD106 targeting specifically the first 6 amino acids, ACC-001 targeting the first 7 amino acids, AD01 and AD02 mimicking the first 6 amino acids, UB-311 targeting the first 14 amino acids, Lu AF20513 targeting the first 12 amino acids, and ACI-24 targeting the first 15 amino acids with a tetra-palmitoylated peptide.^38^ AD03 targets Aβ that is N-terminal truncated and pyroglutamated.^38^ V-950 targets multivalent Aβ.^38^

Median Aβ antibody titers in human trials of ADvac40 reached 810 after the third vaccination (given at week eight).^40^ Median Aβ antibody titers in phase IIa trials of CAD106 reached a maximum (at 8 weeks, 2 weeks after the third injection) of about 40–50 units (in reference to rhesus monkey reference serum), and a sustained level (at 122 weeks, 30 weeks after the last injection) of 23–24 units.^41^ In a phase IIb trial of CAD106, mean Aβ antibody titers reached a maximum (at 8 weeks, 2 weeks after the second injection) of about 40–90 units, and remained between about 20 and 120 units for the rest of trial.^42^ Geometric mean Aβ antibody titers in human trials of ACC-001 reached a maximum (after seven injections over approximately 3 to 3-1/2 years) of at least 40–200 times baseline values.^43^ Mean Aβ antibody titers in human trials of AD02 were 34–72 times baseline values after three injections.^44^ Mean Aβ antibody titers in human trials of UB-311 peaked at 50 times baseline values 4 weeks after the last vaccination at week 16.^42^ Among study arms with at least five subjects in a human trial of V-950, geometric mean Aβ antibody titers at 7 months were 0.74–2.7 times baseline values.^45^ After enrolling three patients, clinical trial NCT03819699 of Lu AF20513 was terminated.^46^ Human trials of ACI-24 have not finished, but the highest measured mean Aβ antibody titers in a mouse study of the equivalent vaccine DS-01 were about 100–200 times baseline values.^47,48^

ADvac40 did not significantly change levels of plasma Aβ_1–40_.^40^ The median concentration of plasma Aβ_1–40_ in phase IIa trials of CAD106 reached a maximum of approximately two to three-fold of the baseline level (at 57 weeks, 1 week after the fourth injection), and maintained nearly that level through week 122.^41^ In a phase IIb trial of CAD106, median concentration of plasma Aβ_1–40_ reached a maximum of approximately 3 times the baseline level (at 38 weeks, 2 weeks after the fifth injection), and maintained a level about double the baseline through week 90, 30 weeks after the last injection.^42^ ACC-001 (pooling results from all three doses) raised least-squares mean plasma Aβ_x-40_ by 84%.^49^ The effect of AD02 on Aβ levels was not reported.^44^ UB-311 increased mean plasma Aβ_1–40_ by 30% at 16 weeks, 4 weeks after the third injection.^50^ The effect of V-950 on Aβ levels was not reported.^45^ Human trials of ACI-24 have not finished, but a mouse study examined the effect of the equivalent vaccine DS-01 on plasma Aβ_1–40_ levels, finding no significant change.^47,48^

Instead of directly injecting an antigen protein, one can inject DNA which cells will transcribe into RNA and then translate into the antigen. A DNA vaccine against Aβ42 trimers reduced plaques and tangles in transgenic mice, perhaps by targeting, in part, Aβ oligomers.^51^

Tau vaccines in human trials include AADvac1, whose antigen is amino acids 294–305 of the 4R form of tau, and ACI-35, whose antigen is a peptide containing pS396 and pS404.^52^ AADvac1 induced an immune response against recombinant pathological tau in 29 of 30 people tested and is now in a phase II trial in AD.^52^ ACI-35 was tested in 24 AD patients in a phase 1b trial, and the related vaccine, ACI-35.030, will be tested in a phase 1b/2a trial.^52,53^

### 3.2. Therapeutic monoclonal antibodies for AD

In addition to traditional active immunization using detoxified antigen as the drug, passive immunization using ex-vivo-generated antibodies is also used for immunotherapy. Passive immunotherapy has potential advantages over vaccination: control over the dose, control over the detailed form of the antibodies, and ability to target an epitope to which it is difficult to generate an appropriate immune response by vaccination. Passive immunization has proven successful in the clinic for various diseases including autoimmune disorders, cancers, and transplant rejections.^54^ Since the advent of hybridoma technology generating mouse monoclonal antibodies in 1975, the idea of using antibodies as a therapeutic agent developed and advanced rapidly.^54^ Although it was recognized initially that mouse antibodies may possess therapeutic limitations (associated with immunogenicity, lack of effector function and short serum half-life), these barriers were eventually overcome by the advent of antibody chimerization and humanization technologies around the mid-1980s.^54^ In 1986, the US Food and Drug Administration approved the first therapeutic antibody: muromonab, which specifically targets CD3 protein for the treatment of acute transplant rejection.^55^ Later, as the field of therapeutic antibody development flourished, treatment options have emerged for autoimmunity, cancer and inflammatory diseases.^55^ Advances in the therapeutic antibody industry were propelled by the success of infliximab, adalimumab, trastuzumab, bevacizumab, and rituximab.^55^

As the treatment of APP transgenic mouse models of AD with therapeutic antibodies to Aβ showed significant reduction in the level of brain Aβ and reversed the memory deficits observed in the object recognition task and Morris water maze, passive immunization has been tested in clinical trials for treatment of AD using the following antibodies: bapineuzumab, AAB-003, GSK933776, solanezumab, ponezumab, BAN2401, gantenerumab, aducanumab, LY3002813, crenezumab, SAR228810, and MEDI1814.^29,56^ They target different epitopes of Aβ and show different preferences for binding Aβ monomer, oligomer, or fibrils (Figure 1).^56,57^ They also belong to different IgG classes, which stimulate different types of immune responses.^56^

Epitopes of several of these antibodies are simple fragments of the Aβ peptide.^57^ Bapineuzumab recognizes Aβ1–5 and binds Aβ monomers and fibrils better than oligomers.^57^ The first clinical trial of an antibody for treating AD used bapineuzumab. There was no change in cerebrospinal fluid (CSF) Aβ levels and no overall change in the worsening of cognition, and testing of both bapineuzumab and an improved version, AAB-003, were stopped.^29,56,58^ GSK933776, which recognizes the N-terminal region of Aβ and binds all forms of Aβ, raised CSF Aβ; testing in AD was stopped.^56,57,59^ In AD, solanezumab, whose epitope is the middle region of Aβ and which binds monomers more than oligomers or plaques, increased CSF Aβ but did not affect the rate of decline in cognition, though it did appear to slow the decline in cognition in the subset of AD patients who had mild AD.^21,29,56,57,60^ However, a subsequent trial in prodromal AD showed no effect on cognitive decline.^61^ Ponezumab binds the C-terminal of Aβ but did not affect CSF Aβ or decline of cognition in AD.^56,57,62^ MEDI1814 also binds the C-terminal of Aβ; initial clinical tests are ongoing.^56,57^

Epitopes of some therapeutic Aβ antibodies are conformations of Aβ.^57^ BAN2401, which binds protofibrils more selectively than fibrils and much more specifically than Aβ monomers, may have raised CSF Aβ and decreased brain amyloid while slowing decline in mild cognitive impairment (MCI) and mild AD, and a larger trial is ongoing to confirm these findings.^57,63,64^ Gantenerumab recognizes two epitopes on Aβ and binds Aβ monomers, oligomers, and fibrils.^57^ It decreased amyloid in brains of AD patients but did not significantly slow cognitive decline, although subgroups (high dose or fast progressors) seemed to benefit; therefore, gantenerumab is now being tested at higher doses.^64^^–^^66^ Monoclonal antibodies are often developed in mice, but aducanumab was developed from healthy elderly people, on the theory that they may produce antibodies that partially protect against AD.^64,67,68^ In initial trials, aducanumab, whose epitope is Aβ3–6 but which preferentially binds oligomers and fibrils over monomers, reduced amyloid and slowed cognitive decline, but the antibody failed to reduce cognitive deterioration in larger, longer studies.^57,64,67^^–^^70^ LY3002813, or N3pG, targets Aβ whose N-terminal two amino acids are removed and whose third amino acid is cyclized into pyroglutamate (pGlu-3 Aβ), making it more amyloidogenic and neurotoxic.^56,71,72^ N3pG decreased brain amyloid and is being tested in a larger trial.^56,64^ In trials of crenezumab, which also targets pyroglutamate Aβ, Aβ oligomers were reduced by a median of 43% (with intravenous delivery) or 48% (with subcutaneous delivery) in CSF, and the highest dose may have raised CSF Aβ and decreased brain amyloid while slowing decline, particularly in the milder AD patients.^56,57,73,74^ Larger trials were conducted; however, those trials were halted in early 2019 because interim analysis indicated that cognitive decline was unlikely to be slowed.^75^ Initial clinical testing of SAR228810, which binds a conformational epitope on protofibrils, is ongoing.^56,57^

None of the passive immunotherapy trials has yet demonstrated a statistically significant slowing of cognitive decline in the primary outcome analysis of the whole patient group tested. Possible reasons for this failure include:^29,56,72^

1. The dose of antibodies may be too low.
2. The antibodies may not reach Aβ.
3. The antibodies may not induce removal of Aβ from the brain.
4. The antibodies may not induce removal of the relevant forms of Aβ.
5. The trials may remove Aβ too late in the disease to alter cognitive decline.
6. Removal of Aβ may not alter cognitive decline.

In recent years, the first factor was found to be a problem with some antibodies, therefore doses in several trials have been increased from decigrams initially to grams in later trials.^64^ Some antibodies, such as many recognizing conformational epitopes, did demonstrate access to Aβ and its removal from the brain;^64^ these antibodies tended to exhibit more promising clinical trial results, perhaps because oligomers or protofibrils may be more toxic than monomers,^57,72,76^ and perhaps because removing monomers from amyloid dissociates plaques, releasing toxic oligomers, like a collapsing Jenga tower.^32^ Plaques might act like dumps in which toxic waste is stored in barrels; it may be safer to leave them undisturbed than to attempt to dismantle and remove them, during which process waste could be released and strewn around. Removing pGlu-3 Aβ may be more effective that targeting other forms of Aβ.^56,71,72^

Several trials showed the strongest trends toward protection by passive immunotherapy in the mildest cases, suggesting that Aβ removal may help only in early stages of AD, perhaps even before any cognitive change but after some biomarkers have changed, such as CSF Aβ42 level, which falls a quarter century before dementia onset.^61,73,77^^–^^79^ Therefore, trials have been started in cognitively normal people who have mutations that will later cause early-onset AD (Alzheimer’s Prevention Initiative [API] and Dominantly Inherited Alzheimer Network-Trials Unit [DIAN-TU]) or who have PET scans showing amyloid in the brain (Anti-Amyloid Treatment in Asymptomatic Alzheimer’s Disease [A4] trial).^29,65,80,81^

Several passive immunotherapy trials have targeted tau. Antibodies RO7105705 and LY3303560 have reached Phase 2 trials in AD, and 8E12, BIIB092, JNJ-63733657, UCB0107, and BIIB076 are in Phase 1 trials in AD or healthy volunteers.^52^

## 4. Role of Aβ

The Aβ passive immunotherapy clinical trials may have failed because they were too late in the disease process, but another possible reason for failure is that the entire approach of reducing Aβ might be wrong. There is evidence for many potentially causative pathways in AD, and many facts contradicting a central etiological role of Aβ,^82^^–^^85^ including the following:

(1)Mutations in APP, PS1, or PS2, which increase Aβ accumulation, cause only a tiny portion of all AD cases, and Aβ might not cause other AD cases.^83^

(2)Aβ amyloid accumulates in other neurodegenerative diseases and in non-demented elderly.^83^

(3)Amyloid plaques do not correlate as closely with dementia as do neurofibrillary tangles or synapse loss.^83^

(4)Removing amyloid from AD patients in clinical trials has not significantly slowed the progression of dementia.^85^

Though there are counter-arguments supporting Aβ as important in AD – (1) in complex diseases, high penetrance mutations only cause a small proportion of cases yet give useful information on disease pathways; (2) perhaps Aβ is toxic only in some circumstances, just as atherosclerosis only causes heart attacks in some cases; (3) Aβ might aggregate tau and harm synapses, which then cause dementia, making Aβ correlate less directly with dementia than do tangles and synapse loss; (4) removing Aβ might only slow AD before Aβ triggers self-propagating tau aggregation, as quitting smoking only reduces lung cancer before tumor initiation – it is certainly important to constantly re-evaluate hypotheses based on available data, to cast a wide net for new data, and to keep thinking outside the box, where many of our previous advances in medicine were found. As the immune system patrols the body on a continuous search for pathogens, digesting molecules and testing them for promotion to the status of antigens, we should vacuum widely and chew on clues to evaluate their worth as hypotheses for pursuit.

This metaphor is relevant in another way: the important role of immune response in AD.^86^ For decades, Ruth Itzhaki and others have accumulated evidence for the involvement of the immune system in AD.^87^^–^^90^ Brain infection by viruses, fungi, or bacteria induces secretion of Aβ, which sticks to the microbe or virus and forms aggregates around it, encasing it.^86,88,90^^–^^94^ Viral DNA or bacteria have been found inside amyloid plaques.^86,88,91,93,95^ Aβ activates an immune response, including stimulation of microglia, inflammation, release of cytokines, complement activation, and further secretion of Aβ itself, which may explain the localized accumulation of Aβ in amyloid plaques and the chronic nature of AD.^87,88,90,95^^–^^98^ Animals expressing Aβ survive infections better than APP-knockout animals.^24,90,93^ Thus, Aβ may function as part of the innate immune system to protect against infection.^88,90,91,93,95,99^ Brain infections by some agents, particularly the herpesviruses herpes simplex virus 1 (HSV1), HSV6A, and HSV7, may trigger AD.^86,88^^–^^91,95,96,98^^–^^100^

Another issue raised by the immune role of Aβ is the potential to prevent or treat AD by anti-infective therapies. To have a meaningful effect, this would require identifying the major infectious agents leading to AD. Evidence for herpesviruses suggests the potential benefit of developing HSV vaccines or of testing existing antiviral drugs, such as aciclovir, which is used to treat HSV encephalitis.^91,95,99,101^ Clinical trials of valaciclovir, a prodrug of aciclovir, are ongoing for the treatment of AD.^102,103^ Several HSV vaccines have been tested unsuccessfully, but others are being developed.^104^

## 5. Future of AD therapy

AD occurs late in life because it develops slowly, implying that it is caused by relatively subtle changes compared to quickly developing diseases, such as some cancers, infections, or childhood illnesses. Developing treatment that blocks these subtle changes without interfering with normal pathways is relatively challenging. Thus, it is not surprising that attempts at treating or preventing AD have not yet succeeded.

Nevertheless, the potential for AD treatment has increased after recent advances in understanding the possible natural function of Aβ in the immune system and in improving the design of passive immunotherapy and vaccines for AD (Figure 1). Antibodies to Aβ and drugs to treat brain infections should be fully investigated. Results with an early trial of aducanumab looked promising, perhaps because the antibody targets oligomers over monomers, decreasing neurotoxicity.^67^^–^^69^ But larger trials in mildly symptomatic AD did not show clinical benefit, suggesting several possible alternative explanations, including the following: Aβ might not be a useful target, treatment too late in the disease process might not work, oligomers might not be a superior target to monomer, or aducanumab might not clear oligomers efficiently enough.^68,70^ Genetic, biochemical, and pathology evidence supports Aβ, and particularly its oligomers, as important in AD, while the many AD clinical trials that have failed were in symptomatic patients.^70^ These are consistent with the second explanation, though others remain possible.

Compared to passive immunotherapy, vaccines have several disadvantages. Vaccines depend on some degree of consistency of the immune response of each individual, but people are heterogeneous. The characteristics of antibodies induced by vaccines are limited by the human immune system and cannot, for example, include artificial modifications which therapeutic monoclonal antibodies might be given to optimize their effectiveness. Monoclonal antibodies can be generated from antigens that would be difficult to store and distribute as a vaccine or that induce side effects that would be intolerable for a vaccine. Antibodies are now being tested for AD at high doses, often several grams, and vaccines may not be able to generate such a high level of antibodies. AD patients are usually elderly, and the weak immune response in old people may require a strong adjuvant, but the resulting inflammatory side effects may be unacceptable.^32^

Despite these problems, vaccines have some important advantages over passive immunotherapy or anti-infective drugs. If anti-infectives or antibodies to Aβ prove effective, they will need to be given multiple times, possibly for life, while vaccines might require few doses, and they tend to be much cheaper than therapeutic monoclonal antibodies.^21,32^ In the United States, it costs about US$2000 to fully vaccinate a child to the age of 18, while it costs about 100 times as much for treatment with just one of the top nine biologicals.^105,106^ Passive immunotherapy will be unaffordable to the vast majority of AD patients, who will increasingly be in poorer countries as life expectancy rises there.^107^ When antibody drugs go off patent, competition from generic versions (biosimilars) could drive prices down, though so far costs remain high (only 35–50% less in Europe and only 15% less in the United States).^105,108^ To make AD treatment affordable to all patients, lower-cost therapies are necessary.

Aside from expense, vaccines might offer biological benefits. People naturally make neuroprotective antibodies (nAbs) against Aβ, and these decrease with age, possibly explaining some of the rise in AD with age.^32^ These human nAbs preferentially recognize oligomers more than monomers and, when injected into mice, improve cognition without decreasing plaques, possibly by removing soluble neurotoxic oligomers without producing more by disrupting plaques.^32,109^ Some nAbs may be IgM, which can hydrolyze Aβ; by contrast, passive immunotherapy generally uses only IgG.^32,110^ Vaccination might be used to induce such nAbs, which may be difficult to create ex vivo, although aducanumab was produced from human nAbs.^32,69^ Epitopes could be conformations found in oligomers but not monomers; though more challenging to design and stabilize such antigens for vaccines than to use simple peptide fragments, this should be feasible.^32,111^

Therefore, in the long term, AD prevention might come to rely on vaccination, whether targeting Aβ or tau or key infectious agents, or combinations of those. As Dennis Selkoe and John Hardy wrote in 2016: “Only 1–2 trials of active Aβ vaccines are underway at this writing, but this approach clearly deserves more study, as the polyclonal antibody response may prove beneficial, and the cost and logistics of distributing passively administered monoclonal antibodies several times per year to the world’s AD population are daunting.”^21^
